# The value of metabolic surgery for patients with obesity and type 2 diabetes in Romania

**DOI:** 10.3389/fnut.2025.1624280

**Published:** 2025-10-07

**Authors:** Gábor Kovács, László Lorenzovici, Cătălina Poiană, Adriana Florinela Catoi, Ciprian Duta, Daniel Vasile Timofte, Marius Florin Coros, Sándor Siménfalvi, Szabolcs Farkas-Ráduly, Tamás Török, Andrea Timea Jakab, Andrea Kacsó, László Nagy, László Imre, Dávid Nagy, Catalin Andu Copaescu, Bogdan Cristian Pana

**Affiliations:** ^1^Syreon Research Institute, Budapest, Hungary; ^2^Syreon Research Romania, Târgu Mureș, Romania; ^3^Faculty of Economics and Business Administration, Babeș-Bolyai University, Cluj Napoca, Romania; ^4^Institutul Național de Endoscrinologie C.I. Parhon, Bucharest, Romania; ^5^Pathophysiology Department, Faculty of Medicine, "Iuliu Hatieganu" University of Medicine and Pharmacy, Cluj-Napoca, Romania; ^6^University of Medicine and Pharmacy "V. Babes", Timișoara, Romania; ^7^Grigore T. Popa University of Medicine and Pharmacy, Iasi, Romania; ^8^G.E. Palade UMPhST of Târgu Mures, Târgu Mureș, Romania; ^9^Medicode SRL, Târgu Mureș, Romania; ^10^Ponderas Academic Hospital, Bucharest, Romania; ^11^Department of Public Health and Management, University of Medicine and Pharmacy "Carol Davila", Bucharest, Romania

**Keywords:** metabolic surgery, bariatry, obesity, diabetes, cost effectiveness

## Abstract

**Background:**

Metabolic surgery is a well-established intervention for managing severe obesity and type 2 diabetes, offering significant long-term health benefits. In Europe, procedures such as sleeve gastrectomy and Roux-en-Y gastric bypass have been shown to improve glycemic control, reduce obesity-related comorbidities, and enhance quality of life. Given the high prevalence of obesity in Romania, evaluating the cost-effectiveness of these surgical interventions is crucial for potential public funding inclusion.

**Methods:**

We adapted a Central European type 2 diabetes—metabolic surgery cost-utility model using Romanian epidemiological and healthcare cost data to assess the cost-effectiveness of weight-loss surgery. The model incorporated three Body Mass Index strata (30–34.9, 35–39.9, ≥40 kg/m^2^) and tracked outcomes over a lifetime horizon. Cost and quality-adjusted life years were evaluated for sleeve gastrectomy and Roux-en-Y gastric bypass using two costing methodologies: Diagnosis-Related Group-based reimbursement and micro-costing analysis. Scenario analyses, including threshold analysis, were conducted to validate model robustness.

**Results:**

Obesity surgery was found to be a dominant strategy, yielding both cost savings and improved health outcomes across all Body Mass Index categories. The intervention led to increased life expectancy, reduced diabetes-related complications, and a significant reduction in healthcare costs. Even under conservative cost assumptions, the surgery remained cost-effective, with an Incremental Cost-Effectiveness Ratio (ICER) well within European funding thresholds.

**Conclusion:**

The results support the inclusion of metabolic surgery in Romania’s public healthcare system. Expanding access could reduce long-term healthcare expenditures while improving the quality of life for individuals with obesity and type 2 diabetes.

## 1 Introduction

Metabolic surgery plays a critical role in Europe as an effective intervention for managing severe obesity and its associated comorbidities, with studies demonstrating significant improvements in long-term weight loss, diabetes remission, and overall quality of life (1, 2). Metabolic surgery induces weight loss through a combination of mechanisms that extend beyond the mechanical restriction of food intake. Procedures like sleeve gastrectomy (SG) and Roux-en-Y Gastric Bypass (RYGB) limit stomach capacity and, in the case of bypass, alter nutrient absorption by diverting portions of the digestive tract. These surgeries elicit hormonal changes, such as reduced ghrelin and increased glucagon-like peptide-1 (GLP-1) and peptide YY (PYY) levels, which suppress appetite and improve satiety, often contributing to diabetes remission (3). They also modify gut microbiota, enhancing metabolic efficiency and insulin sensitivity, and may increase resting energy expenditure. Additionally, postoperative behavioral and neural adaptations shift food preferences and eating patterns, further promoting sustained weight loss. Together, these multifaceted changes underline metabolic surgery’s role as a comprehensive treatment for obesity and metabolic disorders (4). Gastric banding is a less-used technique typically resulting in significantly less weight loss and is associated with more complications, predominately the need for band removal due to intolerance (3, 5, 6). These interventions are mostly performed laparoscopically, requiring only small incisions. Recovery time is usually 4–6 weeks (1). Weight loss is expected to occur in the first 2 years after the surgical intervention (2).

Overweight and obesity affect almost 60% of adults and nearly one in three children (29% of boys and 27% of girls) in the WHO European Region (7). A quarter to one third (22.5–31.4%) of adults in Romania are living with obesity (8, 9). In the clinical setting Body Mass Index (BMI) is used for quantifying overweight and obesity, a value calculated by dividing patients’ weight by their height and expressed in units of kg/m^2^. Obesity in adults is defined as a BMI ≥ 30 kg/m^2^, and is age and sex-independent (10). Obesity should be recognized not only as a risk factor for conditions such as type 2 diabetes and cardiovascular disease, but also as a stand-alone, complex, and chronic disease. This dual perspective is crucial: while obesity contributes to a wide range of health burdens, it also demands direct clinical attention and policy interventions aimed at prevention, early detection, and treatment (10, 11). Weight loss is associated with risk reduction for diabetes, being one of the basic components of diabetes prevention strategies (12).

This study is a country-adapted cost-effectiveness analysis using a health-economic simulation model, aimed to adapt an existing Central-European type 2 diabetes cost-utility model (13, 14) to Romania. Its goal is to estimate the cost-effectiveness of metabolic surgery in people with obesity and type 2 diabetes in Romania, using country-specific clinical and economic inputs. While international evidence demonstrates that metabolic surgery is a cost-effective intervention that leads to substantial long-term health and economic benefits (13, 14), including reductions in obesity-related comorbidities, medication use, and healthcare resource utilization, such evidence has not yet been systematically generated for Romania. This represents a critical research gap: decision-makers in the Romanian healthcare system currently lack robust local cost-effectiveness analyses to guide reimbursement and funding decisions. By providing locally validated projections of long-term healthcare savings, this research has the potential to directly support the justification of the initial investment required for reimbursement and inclusion of metabolic surgery within the publicly funded healthcare system.

## 2 Materials and methods

A previously developed health-economic simulation model on patients with obesity and type 2 diabetes was adapted using Romanian epidemiology and cost data to assess the cost-effectiveness of metabolic surgery (13, 14). The main model drivers included decreased HbA1c resulting in improved glycemic status and improved health utility due to avoided acute events, a decrease in BMI resulting in improved base utility and a decrease in DM treatment intensity resulting in decreased costs. HbA1c or hemoglobin A1c is a measure of chronic hyperglycemia (11). Three BMI strata were separately modeled: 30–34.9, 35–39.9, and ≥40 kg/m^2^. BMI rebound in the 1st–5th postoperative years (after BMI nadir) and HbA1c rebound in the 1st–10th postoperative years (after HbA1c nadir) were assumed in the simulation. A lifetime horizon was used. Outcomes and costs were captured in 6-month cycles. The DM model incorporated a decision tree obesity-specific module on the possible treatments: no surgery, SG surgery, RYGB surgery, and the possibility of conversion from SG to RYGB due to gastroesophageal reflux disease (GERD) or weight rebound. SG/RYGB intervention ratio was set at 60/40. DM complications were simulated by 10 sub-models including stroke, ketoacidosis, ischemic heart disease, hypoglycemia, macular edema, retinopathy, peripheral neuropathy, peripheral vascular disease, foot ulcer, and nephropathy. The model allows the simulation of multiple complications simultaneously for the same patient. The costs and disutility of the complications after SG/RYGB were considered.

All costs of preoperative medical examinations were included in the first model cycle. Metabolic surgery costs included the cost of interventions, postoperative routine care, type 2 DM treatment, and cost of adverse events, including in the first half year minor bleeding, major bleeding, intestinal leak, intestinal perforation, intestinal obstruction, internal hernia, incisional hernia, and abdominal abscess. Equipment cost for surgical interventions was set at €3,555 and was included in the cost calculation. Long term complications, occurring from the second half year included gastro-esophageal reflux, Barrett esophagus, and esophageal cancer.

Model outcomes were survival and quality of life (QoL), incidence of complications, progression of physiological variables, and results for cost-effectiveness analysis. Incremental cost-effectiveness ratio (ICER) was used to quantify the cost-effectiveness of metabolic surgeries compared to the comparator treatment (conventional care of diabetes, i.e., non-surgical treatment of obesity with T2DM, including oral anti-diabetics, insulin therapy and GLP-1 receptor agonists). Lifestyle modifications and other medical treatments like statins, angiotensin-converting enzyme inhibitors, angiotensin 2 receptor blockers, beta-blockers were regarded as sunken cost and effect, i.e., the same effect on both arms of the intervention.

In case the simulation resulted both in cost savings and QALY gains, metabolic surgery was labeled as the “dominant” option over conventional care.

The main principle of the model adaptation was to keep the original structure—the decision tree combined with Markov sub-models. Patients living with obesity (BMI ≥ 30 kg/m^2^) and having type 2 diabetes entered the model at the age of 45. No changes in the structure of the sub-models were made.

Where local data were unavailable, validation of the previously published data (13, 14) was used by key local experts in the field of metabolic surgery.

Local cost calculations for surgery were based on two different methodologies: diagnosis-related group (DRG) and micro-costing. The DRG-based calculation used detailed hospital DRG claim reports and accounting records and statements.

The DRG-based cost calculation for metabolic surgery interventions incorporated detailed hospital DRG claim data and accounting records. Procedures were identified through RO-DRG v1.1 codes: J03902 (Gastric reduction, representing sleeve gastrectomy) and J03903 (Gastric bypass, representing Roux-en-Y gastric bypass). Both procedures were grouped under DRG category K1040 (“Major interventions in obesity”), assigned a relative value of 1.5689. Costing utilized a weighted average fee of €353.57 per DRG point, reflecting variations in DRG point values across Romanian hospital types. The reimbursement rate for DRG group K1040 was set at €554.71, with an additional salary compensation adjustment of +103.35%. Combining these components, the total base-case DRG-based intervention cost was estimated at €1,128. This approach integrated procedural coding, DRG classification, and financial adjustments to derive the final cost estimate.

Micro-costing: Estimation of hospital costs for T2DM complications via micro-costing was based on cost types defined in the standard case-level hospital controlling methodology (15, 16). Micro-costing allowed us to differentiate between the expenses of RYGB and SG. We used hospital claims reports of nine Romanian hospitals from 2023 including two clinical institutes, two clinical hospitals and five county hospitals as the source of micro-costing. Included hospitals discharged a total number of 325,614 cases in 2023 from a total of 3,223,563 cases discharged nationally, representing 10.10%. Average daily care costs by department (ACC dept) and in intensive care (ACC IC) were calculated. Overhead was calculated and added to the costs. Average length of stay in departments (ALoS dept) and in intensive care (ALoS IC) was estimated for each simulated T2DM complication. The following formula was used for model input cost estimations:

ACCdept×ALoS dept+ACCIC×ALoSIC.

Intervention-specific cost differentiation was applied due to variations in surgical duration. For operating room (OR) use, costs incorporated the average OR maintenance cost multiplied by the average length of each intervention (SG, RYGB, surgical management of T2DM complications). This refined approach ensured cost estimates reflected both procedural complexity and time-dependent OR expenses. Length of stay on ward of cases with T2DM and obesity complications are shown in Table 1.

**Table 1:** ALoS on ward of obesity and T2DM complications.

ALoS (days)	Average	SD
Stroke	7.87	9.30
Ketoacidosis	5.81	4.54
Ischemic heart disease	7.26	22.38
Hypoglycemia	4.85	3.89
Macular edema/retinopathy	3.82	3.16
Peripheral neuropathy	7.17	4.23
Peripheral vascular disease	6.18	8.18
Foot ulcer	6.73	7.21
Nephropathy	6.23	5.45

ALoS, average length of stay; T2DM, type 2 diabetes mellitus; SD, standard deviation.

For the costs of medicines, we used expert inputs on market shares of the applicable medications (validated by international data sources), while the final costs were calculated using CANAMED published prices using a weighted average methodology (17).

An annual rate of 3.7% was used for cost and utility discounting, as no official discounting rate was published for Romania. A currency conversion rate of 4.9465 RON/€ was used as the 2023 average annual exchange rate (18).

In case of missing local data for model input, we used the inputs from the original model publication with local expert validation as the best available data.

## 3 Results

### 3.1 Base case analysis of cost-effectiveness: based on DRG costing of the surgeries

We investigated the following BMI/HbA1c strata: BMI 30–34.9/HbA1c 9.5%; BMI 35–39.9/HbA1c 8.3%; BMI 40–50/HbA1c 7.7%. These initial values corresponded to the baseline population characteristics that had been applied in the methodology paper the Romanian analysis was based on (14).

**Figure 1:**
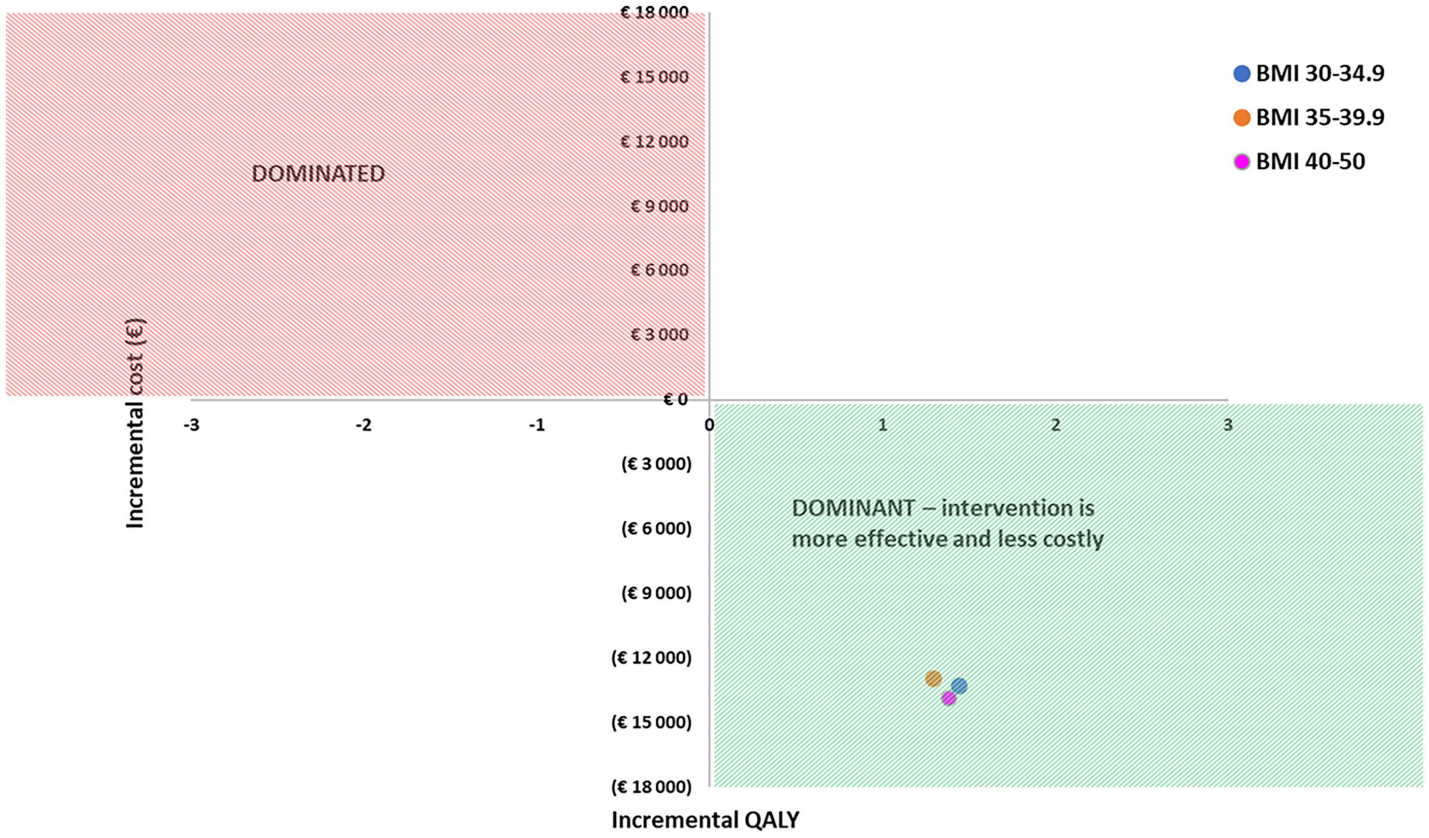
Cost-effectiveness analysis.

Metabolic surgeries were cost-saving and resulted in improvement in health outcomes as well in all three BMI strata (Figure 1). In case of BMI > 40, even at a value of 7.7% for HbA1c (a value below 5.7 is considered as normal) the intervention was still cost-effective (Table 2).

**Table 2:** Base case analysis by BMI categories.

Scenario	Comparator	Metabolic surgery	∆
BMI: 30–34.9 kg/m^2^, HbA1c: 9.5%			
Cost	€62,457	€49,210	€13,247
QALY	7,024	8,464	1,440
BMI: 35–39.9 kg/m^2^, HbA1c: 8.3%			
Cost	€61,641	€48,732	€12,909
QALY	6,925	8,218	1,293
BMI: 40.0–50 kg/m^2^ HbA1c: 7.7%			
Cost	€62,759	€48,905	€13,854
QALY	6,378	7,759	1,381

BMI, body mass index; HbA1c, hemoglobin A1c; QALY, quality adjusted life years.

In all three BMI/HbA1c strata metabolic surgery increased life expectancy (Table 3) and delayed T2DM complications thus offsetting and reducing costs.

**Table 3:** Mean life expectancy and disease-free survival time of T2DM complications (years) by BMI categories.

BMI categories	Comparator	Metabolic surgery	Difference
BMI 30–34.9, HbA1c 9.5%			
Life expectancy	27.7	29.1	1.34
PFS stroke	21.6	23.8	2.17
PFS myocardial infarction	23.5	26.3	2.82
PFS renal transplant	27.6	29.0	1.33
PFS blindness	19.5	22.2	2.75
PFS lower limb amputation	19.9	21.4	1.51
PFS any disease	12.0	14.4	2.45
BMI 35–39.9, HbA1c 8.3			
Life expectancy	28.2	29.2	1.02
PFS stroke	22.3	24.1	1.73
PFS myocardial infarction	24.4	26.6	2.21
PFS renal transplant	28.1	29.1	1.02
PFS blindness	20.8	22.7	1.91
PFS lower limb amputation	20.5	21.7	1.20
PFS any disease	12.9	14.8	1.94
BMI 40–50, HbA1c 7.7			
Life expectancy	28.4	29.2	0.89
PFS stroke	22.7	24.2	1.48
PFS myocardial infarction	24.8	26.8	1.95
PFS renal transplant	28.3	29.2	0.89
PFS blindness	21.5	22.9	1.43
PFS lower limb amputation	20.8	21.9	1.03
PFS any disease	13.5	15.0	1.57

BMI, body mass index; HbA1c, hemoglobin A1c; PFS, progression/disease free survival.

### 3.2 Scenario analysis

#### 3.2.1 Scenario A: cost-effectiveness using micro-costing data for surgery

According to the costing analysis (Table 4), there is a notable difference in actual costs between the two surgical approaches. We found that micro-costing-derived expenses substantially exceed current DRG tariffs for both SG and RYGB. This indicates that existing reimbursement rates for these procedures fail to cover hospital expenditures. The cost-utility analysis was rerun using micro-costing data, incorporating a weighted average cost for both interventions while maintaining the original utility values (unchanged in Table 3).

**Table 4:** Cost comparison of surgeries—DRG vs. micro-costing methodology.

	Costs calculated by micro-costing			
	RYGB		SG	
	Base DRG	Micro-costing	Base DRG	Micro-costing
Costs	€4,683	€6,870	€4,683	€5,301
Ratio of use*	40%		60%	

SG, sleeve gastrectomy; RYGB, Roux-en-Y gastric bypass; DRG, diagnosis related groups; *Based on results of a targeted literature review (TLR) on SG vs. RYGB intervention ratio performed without geographic restriction (53–61). Romanian data based on a limited number of interventions show that SG/RYGB ratio is similar but slightly higher.

#### 3.2.2 Scenario B: threshold analysis using increasing surgical cost data

**Figure 2:**
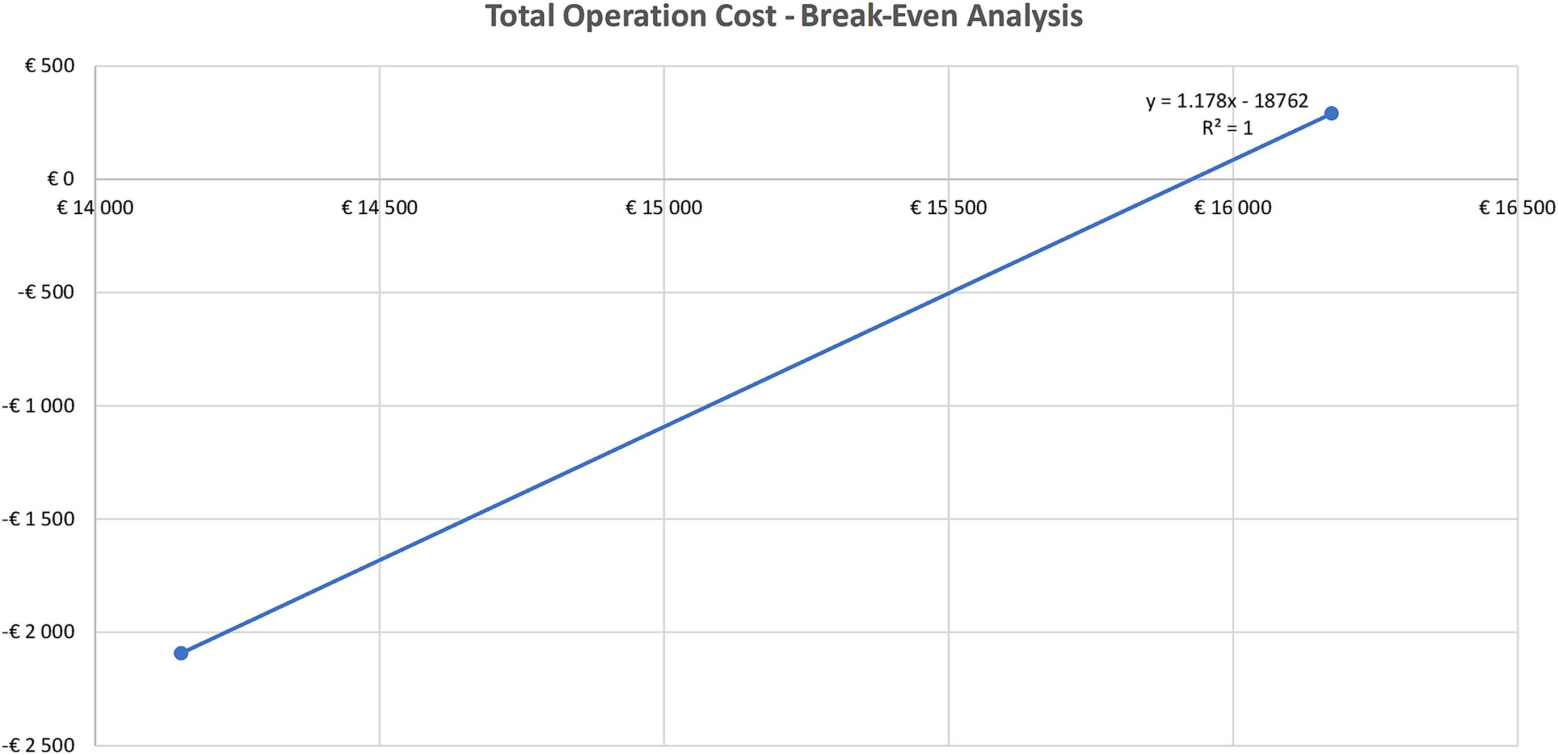
Break-even analysis of total operation cost.

To assess the robustness of the data we conducted a threshold analysis to determine the break-even cost of surgery, i.e., the point at which metabolic surgery is not cost-saving anymore. As anticipated the higher operation costs resulted in less savings (Table 5). It is worthwhile to note that the majority of new health technologies at the time of the introduction in the public funding are not cost-saving, they rather show a better efficacy at a higher cost. Based on the threshold analysis, using a linear regression methodology the break-even cost was at €15,927 (Figure 2) meaning that metabolic surgery is a dominant strategy compared to conventional therapy as long as the surgery cost remains below €15,927. Taking into consideration that the actual cost of operation is €4,683 (based on DRG plus device cost), the cost saving of metabolic surgery is robust.

**Table 5:** Cost-utility analysis outcomes by BMI categories using various hypothetical surgery costs as input parameter—threshold analysis.

	Comparator	Metabolic surgery	∆
Base scenario			
Intervention + device cost = €3,128			
BMI: 30–34.9 kg/m^2^, HbA1c: 9.5%			
Cost	€62,457	€49,210	€13,247
QALY	7.024	8.464	1.440
Scenario I			
Intervention + device cost = €14,151			
BMI: 30–34.9 HbA1c: 9.5%			
Cost	€62,457	€60,365	€2,092
QALY	7.024	8.464	1.440
Scenario II			
Intervention + device cost = €16,173			
BMI: 30–34.9 HbA1c: 9.5%			
Cost	€62,457	€62,746	€289
QALY	7.024	8.464	1.440

BMI, body mass index; HbA1c, hemoglobin A1c; QALY, quality adjusted life years.

#### 3.2.3 Scenario C: analysis with uniform 7% HbA1c levels in all BMI categories

To assess the impact of the HbA1c level on the cost-utility data, we conducted an analysis setting the HbA1c levels at 7% across all BMI categories as a baseline value (Table 6). The rationale for choosing a 7% HbA1c as a fixed value was as follows: For people living with diabetes, the guidelines typically recommend an HbA1c target of less than 7%, although this may be individualized based on patient factors such as age, health status, and risk of hypoglycemia. A HbA1c value below 5.7% is considered normal.

**Table 6:** Cost-utility analysis outcomes by BMI categories using uniform HbA1c levels (7%) as input parameter.

	Comparator	Metabolic surgery	∆
BMI: 30–34.9 kg/m^2^, HbA1c: 7%			
Cost	€58,209	€48,069	€10,140
QALY	7.763	8.68	0.92
BMI: 35–39.9 kg/m^2^, HbA1c: 7%			
Cost	€59,664	€48,109	€11,555
QALY	7.279	8.35	1.07
BMI: 40–50 kg/m^2^, HbA1c: 7%			
Cost	€61,843	€48,489	€13,354
QALY	6.554	7.855	1.3

BMI, body mass index; HbA1c, hemoglobin A1c; QALY, quality adjusted life years.

Based on the cost-utility data even at low HbA1c levels (mild T2DM), metabolic surgery is a dominant strategy vs. conventional therapy, providing a less costly, more effective intervention (Table 7).

**Table 7:** Cost-utility analysis outcomes: DRG vs. micro-costing methodology.

	Savings	
	Base DRG	Micro-costing
BMI 30–34.9 HbA1c: 9.5%	€13,248	€11,624
BMI 35–39.9 HbA1c: 8.3%	€12,910	€11,285
BMI 40–50 HbA1c:7%	€13,855	€12,229

BMI, body mass index; HbA1c, hemoglobin A1c; DRG, diagnosis related groups.

## 4 Discussion

The objective of this cost-effectiveness analysis was to assess whether bariatric surgery represents a cost-effective treatment option for people living with obesity and type 2 diabetes, from the perspective of the public payer. The analysis included individuals aged 45 years and older with a body mass index (BMI) of at least 30 kg/m^2^. It compared the bariatric procedures most commonly performed in Romania with conventional diabetes management. Health outcomes were measured in quality-adjusted life years (QALYs), and cost estimates were based on public payer expenditures. The findings demonstrated that sleeve gastrectomy (SG) and Roux-en-Y gastric bypass (RYGB) not only improved health outcomes but also reduced costs, making them ‘dominant’ treatment options compared to conventional diabetes therapy. This dominance was consistent across all three BMI categories examined.

A 2022 systematic literature review from Ireland highlights that international evidence consistently demonstrates metabolic surgery to be a cost-effective or cost-saving intervention for individuals living with type 2 diabetes and obesity. The included studies, spanning multiple countries, were generally of high quality and robust across sensitivity analyses, though limitations remain regarding long-term follow-up data and uncertainties around the utility values associated with diabetes remission. Importantly, no studies from Eastern European countries were identified, underscoring a critical evidence gap in regions where the prevalence of obesity and diabetes is rising. This absence of regional data limits the direct transferability of international findings to Eastern European healthcare systems and emphasizes the need for locally adapted economic evaluations to guide decision-making and resource allocation (19).

A recent Romanian single-center prospective and case–control study (20) compared laparoscopic gastric plication (LGP) and laparoscopic sleeve gastrectomy (LSG) in terms of weight loss outcomes, safety, and comorbidity improvement. The analysis of 100 patients (50 per group) showed that LSG resulted in significantly greater weight loss starting from 6 months post-surgery, with the largest differences observed at 24 and 36 months. Both procedures were equally effective in improving obesity-related comorbidities and had similar safety profiles and hospitalization durations. LGP may be more suitable for patients with a BMI < 40 kg/m^2^, while LSG offers superior long-term weight loss benefits.

Data from Romanian centers (21) indicate a 65–75% reduction in body mass index (BMI) over a year, with notable improvements in comorbid conditions like type 2 diabetes and hypertension. Statistically significant reductions in HbA1c levels, averaging 2.5%, were observed among patients living with diabetes after surgery, showcasing the metabolic benefits of these procedures.

As overweight and obesity are becoming widespread not only in the high-income but in the low-and middle-income countries as well, there is an increasing need for efficient management (22). Metabolic surgery is a highly effective treatment option for individuals with severe obesity who cannot be successfully treated with non-surgical interventions of weight loss (23). Several obesity-related conditions including type 2 diabetes respond well to metabolic surgery. Our scenario analysis showed that in the case of extreme obesity, even with a mild form of T2DM, metabolic surgery is still cost-effective. Improvement in mobility, overall health, psychological health, and QoL was also reported as a result of the surgical treatment (2).

A Romanian study in 2023 evaluated the QoL of 76 patients following metabolic surgery using two types of questionnaires—SF-36 and the WHO’s Quality of Life-BREF (WHOQOL-BREF) questionnaire. The study reported that out of the 76 respondents, 39.47% underwent gastric bypass surgery (RYGB), 56.57% underwent SG, and only 3.94% underwent single anastomosis duodenum-ileum switch (24). The lowest average scores were found in the vitality subscales (61.13 ± 15.20) and limitations due to mental health (65.78 ± 13.30), which remained consistent across surgery types (24). The subscales with the highest mean values for a particular surgical intervention are physical pain (92.73 ± 14.23), social functionality (89.53 ± 15.65), and limitations due to physical health (87.20 ± 30.06), which are linked to the SG procedure (24).

A 2023 single-center study on the safety and short-term results of LSG, RYGB, laparoscopic gastric plication and intragastric balloon reported a partial remission of diabetes in 25.3% of cases, and total remission in 61.4% of patients (21).

A retrospective pilot study in 2019 analyzed a group of 59 consecutive patients with obesity, which were subjected to metabolic surgery. The study reported BMI, waist circumference, and total body weight decreased by 38, 31, and 41%, respectively 12 months after the intervention versus baseline values (*p* < 0.001). Blood glucose and triglycerides levels decreased significantly as well, by 16 and 37%, respectively (*p* < 0.001). In patients with diabetes, glycated hemoglobin (HbA1c) decreased by 28% and the necessity for antidiabetic medical treatment dropped by 69%. Metabolic surgery improved all components of the metabolic syndrome thus reducing the cardiovascular risk (25).

A study on laparoscopic Roux-en-Y feeding jejunostomy (LRYFJ) for patients with leaks or fistulas after gastric sleeve surgery (GSS) showed promising results. Six patients (four females, two males, average age 37.1 years) underwent the procedure with an average operative time of 127.5 min and a mean duration of jejunal nutrition of 183.83 days. There was no mortality associated with the surgery. Five patients (83.3%) had their fistulas successfully treated with LRYFJ, while one patient’s leak healed spontaneously. The study concluded that LRYFJ is a safe and effective method for providing long-term nutritional support to these patients (26).

To utilize these potential benefits for metabolic surgeries, the introduction of public funding for a well-defined population would be crucial to address the consequences of the growing prevalence of obesity and its associated comorbidities (T2DM, cardiovascular diseases, sleep apnea, and joint disorders). The inclusion of metabolic surgery into public funding requires demonstrating its cost-effectiveness, as it ensures efficient allocation of limited healthcare resources while achieving significant long-term health and economic benefits.

Concerning the cost-effectiveness of metabolic surgery using local cost data, we were able to demonstrate that surgery was a dominant strategy vs. traditional patient care, demonstrated cost saving in all BMI strata of the base case analysis (BMI: 30–34.9 kg/m^2^, HbA1c: 9.5%; BMI: 35–39.9 kg/m^2^, HbA1c: 8.3%; BMI: 40–50 kg/m^2^, HbA1c: 7.7%) with a better quality of life (QoL). Setting the baseline HbA1c at 7% in all the above scenarios, the dominance of metabolic surgery vs. traditional patient care remained.

The cost-effectiveness analysis was primarily performed using DRG-based costs, as these represent the standard reimbursement model within the public healthcare system (27). However, to provide a more comprehensive perspective, the analysis was also conducted using costs derived from the micro-costing method. This approach offers a more precise estimation of actual hospital expenditures by incorporating detailed audit-based cost data, including resource utilization and surgical equipment expenses.

The inclusion of micro-costing-based costs was particularly important to highlight the robustness of the cost-effectiveness findings. Specifically, it demonstrated that metabolic surgeries, such as RYGB and SG, would remain cost-effective—or even financially dominant—under a scenario where the public payer fully covered all hospital-related expenses. This reinforces the economic viability of these procedures in the long term, even under a more comprehensive funding model.

The results of our analysis are supported by analysis outcomes from other countries that consistently demonstrated the cost-effectiveness of metabolic surgeries across Europe by reducing long-term healthcare costs and improving patients’ quality of life. Surgery achieved cost-effectiveness primarily through long-term cost savings on chronic disease management with an average ICER ranging from €5,000 to €20,000 per QALY, well within the usual cost-effectiveness thresholds. The average cost savings are estimated at €15,000 per patient over a decade. The QALYs gained per patient ranged between 1.8 and 6.7, varying with baseline health and surgical approach. Countries with higher healthcare costs (e.g., Switzerland, UK) show greater economic benefits due to higher baseline expenditure on obesity-related care, although Southern European countries (e.g., Spain, Italy) still demonstrated significant QALY gains despite lower healthcare expenditure. The findings strongly support its adoption as a standard intervention for managing severe obesity.

The cost-effectiveness of metabolic surgery in Romania is supported by the available but still limited local data as well, demonstrating substantial healthcare savings from reduced medication use and hospitalizations for obesity-related illnesses (28, 29), resulting in a net healthcare saving of approximately €8,000 per patient annually due to reduced diabetes management costs. The quality of life (QoL) assessments indicate a 60–70% improvement post-surgery, with patients reporting enhanced physical mobility and psychological well-being (30).

Further studies—ideally prospective, observational, and reflective of real-world clinical practice—are needed to strengthen the evidence base and provide decision-makers with reliable information for reimbursement and public funding decisions. Studies analyzing data from existing disease registries would provide especially valuable results, as registries capture large, representative patient populations over extended periods of time. Unlike clinical trials, which are often restricted by strict eligibility criteria, registry data reflect routine clinical practice and real-world variability in patient characteristics, comorbidities, and treatment patterns. Such analyses can provide detailed insights into long-term outcomes, healthcare utilization, and treatment adherence, while also allowing stratification by relevant subgroups such as age, BMI, and disease duration.

To put into context the cost-effectiveness result, based on the Romanian epidemiological data, 13.0% of the adult population has a BMI between 30 and 35 (2.13 million people), 5.8% has a BMI between 35 and 40 (0.92 M lives) and 3.9% has a BMI higher than 40 (0.63 M lives) (31–36). The International Federation for Surgery of Obesity and Metabolic Disorders-European Chapter (IFSO-EC) guidelines consider metabolic bariatric surgery as part of a multimodal approach for adult patients with a BMI ≥ 30 kg/m^2^ (37). Metabolic surgery is “Recommended” for patients between BMI 35–40 kg/m^2^, especially with severe/moderate comorbidities, and “Strongly Recommended” for patients above 40 kg/m^2^ BMI without any conditions. Based on these two categories “Recommended” (around 40% of the 35–40 BMI population) (3, 38–46) “Strongly Recommended” (100% of the >40 kg/m^2^ BMI population)—using European comorbidity data—more than 1 million patients (0.38 + 0.63 million) are eligible for surgery in case of conventional therapy—for various reasons—is not providing the required results. The relatively sporadic Romanian epi data seem to confirm the projected numbers above (47–50).

In recent years, several studies have focused on adapting international health-economic models to local contexts in Central and Eastern Europe. A 2019 study by Lorenzovici et al. assessed the budgetary implications of introducing Ferric Carboxymaltose (FCM) treatment in Romanian hospitals, using a model adapted from an existing German economic evaluation framework. Analysis was conducted from the public payer’s perspective, making it directly applicable to National Health Insurance decision-making (51).

Our study has some limitations. The analysis considered the consequences of T2DM only, although it can reasonably be expected that the taking into account of other metabolic and nonmetabolic consequences of obesity would further increase health gain achieved. The analysis did not include patients living with obesity but without T2DM so very limited conclusion can be drawn regarding this population. The analysis was conducted to three BMI strata separately, but no “mixed BMI” population analysis was performed. The simulation included people with diabetes who were over 45 years old, as this age group is considered the most representative of individuals living with obesity and type 2 diabetes in Romania, according to expert opinion. The whole model population was assumed to be treated with insulin before surgery and some were assumed to continue insulin with reduced doses after surgery (as a conservative estimation following expert opinion). A study limitation is that for certain Romanian input parameters (such as baseline BMI distribution, HbA1c levels, and cost data), sample sizes and measures of variability were not consistently available in the published sources. Where possible, we complemented national epidemiological surveys (e.g., PREDATORR) with expert validation and hospital audit data.

## 5 Conclusion

This cost-effectiveness analysis provided evidence that metabolic surgeries are both cheaper and better alternatives than conventional diabetes treatment resulting in improved health outcomes in the population with obesity. In all three BMI strata base case model results showed a QALY gain of 1.293–1.440 as well as cost savings reaching €13,854 per patient. Metabolic surgery results in long-term cost savings on chronic disease management (e.g., savings in T2DM national health program budget due to decreased medication use and reduction in insulin requirements). Despite these benefits, due to the lack of public funding, access to metabolic surgery remains low and uneven, with urban centers like Bucharest and Cluj-Napoca seeing higher procedure rates compared to rural areas, reflecting disparities in healthcare access and awareness (52). Furthermore, public funding would ensure equitable access to surgery, particularly for low-income populations who are disproportionately affected more by obesity but lack the resources to afford treatment.

Based on the threshold analysis the break-even cost was at €15,927 meaning that metabolic surgery is a dominant strategy compared to conventional therapy as long as the surgery cost remains below this value. The inclusion of metabolic surgery in public funding has budgetary implications, especially under financial constraints. However, clear and transparent legislation, along with evidence-based protocols (e.g., accessibility of surgery to a well-defined patient group), ensure its long-term sustainability.

Challenges remain in expanding access to metabolic surgery to meet the needs of people in Romania who are living with obesity, a population that continues to rise.

The emerging data underscore the transformative role of metabolic surgery in Romania’s healthcare landscape and highlight the need for systemic efforts to broaden its availability.

## Data Availability

The raw data supporting the conclusions of this article will be made available by the authors, without undue reservation.
